# Fluvastatin suppresses hemin-induced cell death, reactive oxygen species generation, and elevated labile iron pool

**DOI:** 10.1016/j.htct.2024.09.2480

**Published:** 2024-11-07

**Authors:** Shion Imoto, Katsuyasu Saigo, Mari Kono, Ayako Ohbuchi, Tohru Sawamura, Yuji Mizokoshi, Takashi Suzuki

**Affiliations:** a Faculty of Medical Technology, Kobe Tokiwa University, Kobe, Hyōgo, Japan; b Life Science Center, Kobe Tokiwa University, Kobe, Hyōgo, Japan; c Seikaen Youriki Center, Akashi, Hyogo, Japan.; d Faculty of Nursing, Himeji Dokkyo University, Himeji, Hyogo, Japan; e R&D Center Asia Pacific, Sysmex Asia Pacific, Asia Green, Singapore; f Faculty of Pharmacological Sciences, Himeji, Hyogo, Japan

**Keywords:** Iron overload, Macrophage, Deferasirox, Statins, Ferroptosis

## Abstract

**Background:**

In transfusion-related iron overload, macrophage/reticuloendothelial cells are the first site of haem-derived iron accumulation. The prevention of haem-induced cytotoxicity in macrophages may represent a target for iron overload treatment. Deferasirox, an oral iron chelator, has been used to treat transfusion-related iron overload however, low adherence to the therapy is an issue. Statins, which are widely used for the prevention of atherosclerotic cardiovascular diseases, also have anti-oxidative and anti-inflammatory effects independent of their lipid lowering ones. Whether statins can suppress hemin-induced cytotoxicity and enhance the cytoprotective effects of deferasirox are important considerations to improve transfusion-related iron overload treatment. This study also evaluated the effects of eltrombopag, a thrombopoietin receptor agonist.

**Materials and methods:**

Human monocytic THP-1 cells were pretreated with statins, deferasirox, and/or eltrombopag, followed by treatment with hemin. Cell viability, reactive oxygen species generation, and the intracellular labile iron pool were measured using flow cytometry.

**Results:**

Fluvastatin and another four statins suppressed hemin-induced cell death, reactive oxygen species generation, and increases in the labile iron pool. Moreover, fluvastatin enhanced the suppressive effect of deferasirox on hemin-induced cell death. The effects of eltrombopag were similar to those of the statins.

**Conclusion:**

The safety of statins is well established. When used in combination with fluvastatin or other statins, the suppressive effects of deferasirox on hemin-induced cytotoxicity in THP-1 cells were amplified. Further research is necessary to see whether statins will act in the same way *in vivo* or in human primary monocytes/macrophages.

## Introduction

Iron overload is a major problem that can negatively affect the clinical outcomes of transfusion-dependent patients.^1^, ^2^, ^3^ Macrophage/reticuloendothelial cells are the first sites of iron accumulation in cases of transfusion-related iron overload (TRIO). Selective accumulation of iron in reticuloendothelial cells is relatively safe and protects parenchymal cells from iron overload.^1^ When the amount of loaded iron exceeds the protective capacity of macrophages, iron accumulates in parenchymal cells and causes organ dysfunction. Therefore, prevention of haem-induced macrophage cytotoxicity may represent a therapeutic target of TRIO.

TRIO is treated with iron chelators. Of three iron chelators, deferoxamine, deferasirox (DSX) and deferiprone, DSX is the most popular choice in Japan.^4^ DSX is administered orally once per day; its efficacy is well established.^5^^,^^6^ However, among adult patients with TRIO, adherence to the DSX treatment regimen is low due to adverse events such as gastrointestinal symptoms and renal dysfunction.^5^ A strategy for dose reduction without lowering the treatment's efficacy is therefore warranted.

Statins are 3-hydroxy-3-methylglutaryl-coenzyme A (HMG-CoA) reductase inhibitors that are used to treat hyperlipidemia and as such their safety and clinical efficacy have been well established in large clinical trials, and statins are widely used for primary and secondary prevention of atherosclerotic cardiovascular disease.^7^, ^8^, ^9^ Apart from reducing plasma cholesterol levels, statins also exhibit pleiotropic properties such as anti-inflammatory and anti-oxidative effects, and neuro- and renal-protective effects, as well as cancer-preventing effects.^10^, ^11^, ^12^, ^13^ Whether statins may also have beneficial effects for TRIO treatment, and whether they might enhance the therapeutic effects of DSX, remain important clinical questions to be answered. If they do prove beneficial, lower doses of DSX used in combination with statins may represent an alternative treatment regimen that could improve adherence.

Eltrombopag (ELT) is a thrombopoietin receptor agonist that is used to treat thrombocytopenia. The potent iron chelating ability of ELT has been reported in recent studies.^14^^,^^15^ ELT and DSX synergistically reduce intracellular iron levels.^14^ Whether ELT is effective in the treatment of TRIO remains an interesting clinical question.

In previous studies, we investigated the mechanisms underlying TRIO in search of better therapeutic strategies.^16^, ^17^, ^18^, ^19^, ^20^ The human monocytic cell line THP-1 is widely used as a model for studying macrophage function. Using THP-1 and hemin as a model of haem-iron-induced macrophage injury, we reported the beneficial effects of DSX on hemin-induced cytotoxicity.^17^^,^^18^^,^^20^ This study examines the effects of statins on hemin-induced cytotoxicity and investigates the effects of ELT, which was expected to exhibit iron chelating properties.

## Materials and methods

### Cell culture, treatment with hemin and other reagents

Human monocytic cell line THP-1 were maintained in RPMI1640 medium supplemented with 10% fetal bovine serum (FBS), according to a previously described protocol.^18^

The cells were washed with phosphate buffered saline (PBS) to remove FBS, suspended in serum-free RPMI1640, and seeded in the wells of a 24-well plate. Statins and/or other reagents for examination were added to the wells and incubated in 5% CO_2_ at 37°C, prior to treatment with hemin. The reagents examined were: atorvastatin, rosuvastatin, pitavastatin (Pita), fluvastatin (Flu), pravastatin (PS), (Cayman Chemical, Ann Arbor, MI, USA), Simvastatin (Sim) (Adipogen AG, Schneckelerstrasse, Füllinsdorf, Switzerland), DSX (Cayman Chemical) and ELT (Chem Scene, Monmouth Junction, NJ, USA). Following the pretreatment, hemin was added to the wells and the plates were again incubated in 5% CO_2_ at 37°C. According to previous studies,^18^^,^^20^ hemin treatment used 20 μmol/L for 90 min for the cell viability assay, and 10 μmol/L for 1h for the reactive oxygen species (ROS) and labile iron pool (LIP) assays

### Measurement of cell death, reactive oxygen species and labile iron pool

Cell death and ROS generation were measured using a previously described protocol.^18^ Briefly, cell death was detected using double staining with annexin-V and propidium iodide, and analyzed using flow cytometry. ROS generation was measured based on the oxidation of 5-(6-)chloromethyl-2′,7′-dichlorodihydrofluorescein diacetate (CM-H2DCFDA), and analyzed using flow cytometry. Furthermore, intracellular LIP was measured using calcein acetoxymethyl ester and analyzed by flow cytometry.^20^

### Statistical analysis

Quantitative data are presented as the mean ± standard deviation. Differences between intergroup pairs were determined using the Student's t-test. Probabilities (*p*-values) of <0.05 were considered statistically significant.

## Results

### Statins suppressed hemin-induced cell death

The effects of six statins, atorvastatin, rosuvastatin, Pita, Sim, Flu, and PS on hemin-induced cell death were examined. Five exhibited suppressive effects at concentrations ranging from 2 to 10 μmol/L. PS was not found to be effective even at doses as high as 100 μmol/L so it was excluded from subsequent tests.

**Figure 1:**
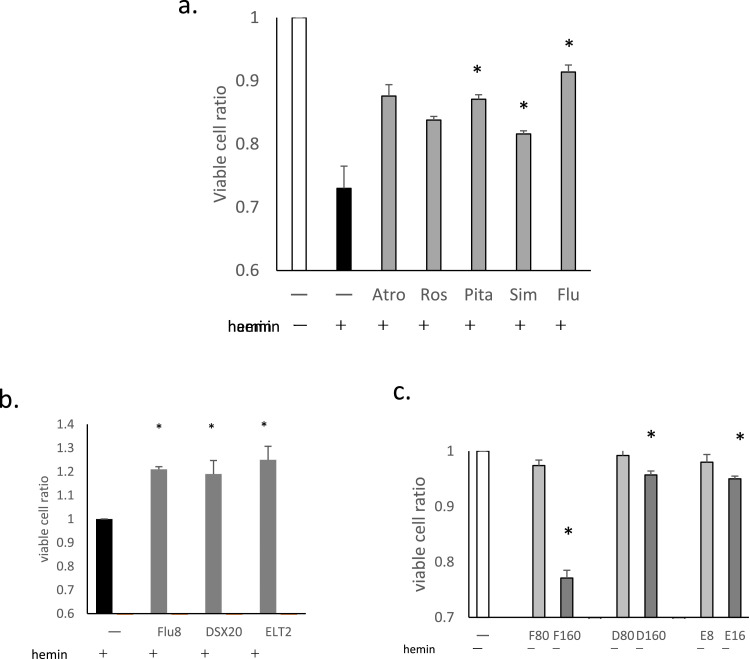
The effects of statins and other drugs on hemin-induced cell death. THP-1 cells were pretreated with statins, deferasirox (DSX), or eltrombopag (ELT), followed by treatment with hemin at 20 μmol/L for 90 min (a-c). Following the treatment, cell viability was evaluated using flow cytometry. (a)The effects of the five statins are presented as ratios of viable cells (%) compared to negative controls (no hemin treatment; the ratio of the white column is 1). All statin pretreatments were at 8 μmol/L for 16h. Data for three representative experiments are presented as means ± standard deviation (SD). *p-value <0.05 determined using a two-tailed unpaired Student's t-test. (b)The effects of fluvastatin (Flu: 8 μmol/L, 16h), DSX (20 μmol/L, 1h) and ELT (2 μmol/L, 1h) are presented as viable cell ratios compared to hemin treatment alone (the ratio of the black column is 1). Data for three representative experiments are presented as means ± SD. (c)The cytotoxicities of Flu, DSX, and ELT without hemin treatment. THP-1 cells were treated with Flu (80 μmol/L [F80] or 160 μmol/L [F160], 16h), DSX (80 μmol/L [D80] or 160 μmol/L [D160], 1h), or ELT (8 μmol/L [E8] or 16 μmol/L [E16], 1h). Cell viability is presented as the ratio of viable cells (%) compared to negative controls (no treatment; the ratio of the white column is 1). Data for three representative experiments are presented as the means ± SD.

Overnight treatment was necessary for the suppressive effects to become apparent. The suppressive effects of the five statins at 8 μmol/L after 16 h of treatment are shown in Figure 1a. Pita, Sim and Flu showed significant effects (*p*-value = 0.0124, 0.0214 and 0.019, respectively), whereas those of atorvastatin and rosuvastatin were not statistically significant (*p*-value = 0.105 and 0.054, respectively). Flu was therefore chosen for further analysis, as it appeared to be most effective.

The suppressive effects of DSX and ELT were then compared to those of Flu. Both DSX at 20 μmol/L for 1 h of treatment, and ELT at 2 μmol/L for 1 h of treatment significantly suppressed hemin-induced cell death (*p*-value = 0.01 and 0.006, respectively), with efficacies similar to that of Flu at 8 μmol/L for 16 h of treatment (*p*-value = 0.013; Figure 1b).

Next, the cytotoxic effects of Flu, DSX and ELT were evaluated. Decreases in viable cell ratios were not observed with Flu up to 80 μmol/L for 16 h, DSX at 80 μmol/L for I h, or ELT at 8 μmol/L for 1 h (Figure 1c). At higher doses, significant decreases in viable cell ratios were observed for all the three reagents.

### Comparison of suppressive effects of fluvastatin, deferasirox and eltrombopag on hemin-induced cell death, reactive oxygen species generation and increases in labile iron pool

**Figure 2:**
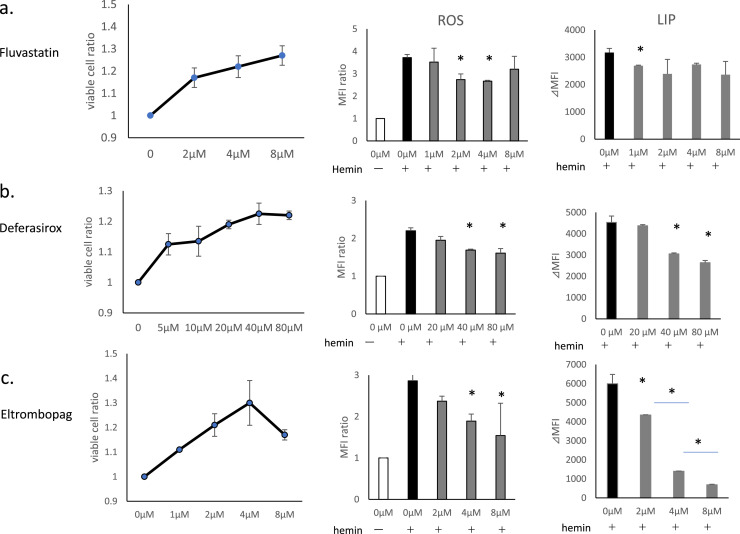
The effects of fluvastatin (Flu), deferasirox (DSX) and eltrombopag (ELT) on hemin-induced cell death, reactive oxygen species (ROS) generation, and increases in labile iron pool (LIP). THP-1 cells were pretreated with Flu, DSX or ELT followed by treatment with hemin. Treatment with hemin was 20 μmol/L for 90 min to evaluate cell death/viability, and at 10 μmol/L for 1h to evaluate ROS and LIP. Cell viability is presented as the ratio of viable cells compared to hemin treatment alone (the ratio of the left lane is 1). ROS levels were evaluated using CM-H2DCFDA (0.5 μmol/L). ROS generation is expressed as the relative peak fluorescence intensity compared to that of the negative control (without hemin treatment, the ratio of the white column is 1 - middle lane). LIP levels were evaluated using calcein acetoxymethyl ester and are presented as ⊿median fluorescence intensity (MFI) between each test sample and that of hemin treatment alone (right lane). (a) Flu: the duration of pretreatment was 16h at concentrations of 2, 4, 8 μmol/L for cell death/viability assays and at 1, 2, 4, and 8 μmol/L for ROS and LIP evaluations. (b) DSX: the duration of pretreatment was 1h at concentrations of 5, 10, 20, 40, and 80 μmol/L for cell death/viability assays and at 20, 40, and 80 μmol/L for ROS and LIP evaluations. (c)ELT: the duration of pretreatment was 1h at concentrations of 1, 2, 4, 8 μmol/L for cell death/viability assays and at 2, 4, and 8 μmol/L for ROS and LIP evaluations. Data of three representative experiments are presented as means ± standard deviation (SD. *p-value <0.05 determined using a two-tailed unpaired Student's t-test.

In addition to cell death, hemin induces intracellular increases in LIP and ROS generation, as has been reported previously.^18^^,^^20^ In this study, we examined the effects of Flu, DSX, and ELT on hemin-induced ROS generation, and increases in LIP, in addition to cell death (Figure 2).

Flu suppressed cell death at 2 μmol/L, and exerted increasing protective effects in a dose-dependent manner up to 8 μmol/L (Figure 2a). Flu suppressed ROS generation at 2-4 μmol/L, but not at 8 μmol/L. Its suppressive effect in terms of increases in LIP was significant at 1 μmol/L (*p*-value = 0.048), but not at higher doses.

DSX suppressed cell death at concentrations as low as 5 μmol/L, and this effect increased in a dose-dependent manner up to 40 μmol/L (Figure 2b). Doses of DSX of ≥40 μmol/L were necessary to suppress ROS generation and increases in LIP.

ELT suppressed cell death at concentrations as low as 1 μmol/L, and this effect increased in a dose-dependent manner up to 4 μmol/L, but decreased at higher doses (Figure 2c); doses ≥4 μmol/L were necessary to suppress ROS generation. ELT suppressed increases in LIP at concentrations as low as 1 μmol/L with this suppression increasing in a dose-dependent manner.

### The effects of combination treatment

**Figure 3:**
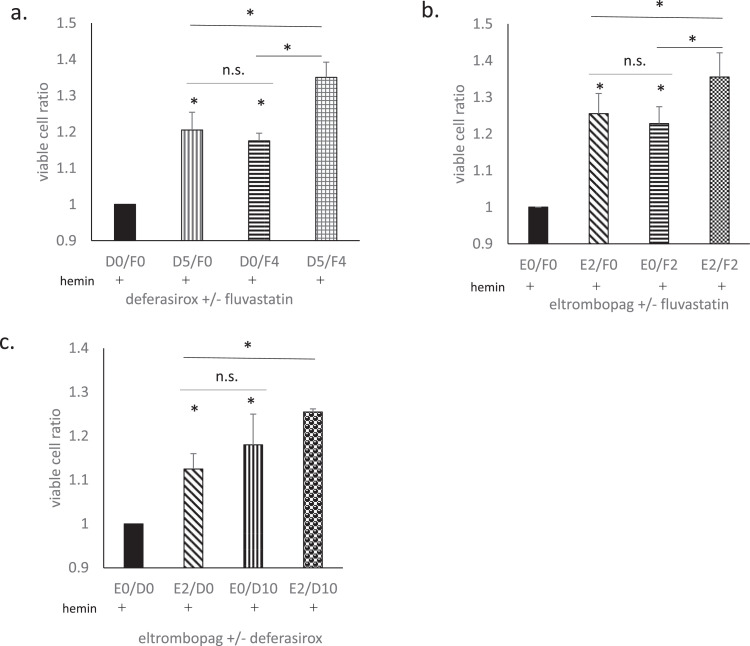
The effects of combination treatments. The effects of combination treatments with fluvastatin (Flu) + deferasirox (DSX), Flu + eltrombopag (ELT), and DSX + ELT were evaluated. Hemin treatment used 20 μmol/L for 90 min. The cell viability is presented as the ratio of viable cells compared to hemin treatment alone (the ratio of the black column is 1). (a)The effect of DSX at 5 μmol/L for 1 h (D5/F0), Flu at 4 μmol/L for 16h (D0/F4), and DSX at 5 μmol/L for 1 h plus Flu at 4 μmol/L for 16h (D5/F4). (b)The effect of ELT at 2 μmol/L for 1 h (E2/F0), Flu at 2 μmol/L for 16h (E0/F2), and ELT at 2 μmol/L for 1 h plus Flu at 2 μmol/L for 16h (D5/F4). (c)The effect of ELT at 2 μmol/L for 1 h (E2/D0), DSX at 10 μmol/L for 1h (E0/D10), and ELT at 2μmol/L for 1 h plus DSX at 10 μmol/L for 16h (E2/D10). Data from three representative experiments are presented as means ± standard deviation (SD). *p-value <0.05 determined using a two-tailed unpaired Student's t-test. n. s., not statistically significant (*p*-value ≥0.05).

Flu, DSX, and ELT were all found to suppress hemin-induced cell death to some degree. Therefore, we examined whether treatment combining two of these drugs could further enhance the suppressive effects (Figure 3).

DSX at 5 μmol/L for 1h (D5/F0) and Flu at 4 μmol/L for 16 h (D0/F4) significantly suppressed hemin-induced cell death. The suppressive effects of DSX and Flu are similar, and the combination of the two (D5/F4) further augmented the total suppressive effect more than either drug alone (*p*-value = 0.00036 and 0.0011, respectively; Figure 3a).

ELT at 2 μmol/L for 1h (E2/F0) and Flu at 4 μmol/L for 16 h (E0/F4) also significantly suppressed hemin-induced cell death. The suppressive effects of ELT and Flu were similar, and the combination of the two (E2/F4) further augmented the total suppressive effect more than either drug alone (*p*-value = 0.035 and 0.00021, respectively; Figure 3b).

ELT at 2 μmol/L for 1h (E2/D0) and DSX at 10 μmol/L for 1 h (E0/D10) also significantly suppressed hemin-induced cell death (*p*-value = 0.0074 and 0.025, respectively). The suppressive effect of DSX plus ELT (E2/D10) was significantly higher than that of ELT alone (*p*-value = 0.03) but not significantly higher than that of DSX alone (*p*-value = 0.1; Figure 3c).

## Discussion

In this study, statins were found to suppress hemin-induced cell death. Flu was also found to suppress hemin-induced ROS generation and increases in LIP, as well as enhanced the suppressive effects of DSX. Anti-oxidative effects of statins have been intensively studied. One explanation is the statin-mediated activation of NF-E2-related factor 2 (Nrf2).^21^^,^^22^ Nrf2 is a stress-response transcription factor that regulates a number of anti-oxidative factors such as haem oxygenase 1 (HO-1) and glutathione peroxidases (GPX), thus exerting anti-oxidative effects.^21^^,^^22^

Ferroptosis is a type of programmed cell death caused by iron-mediated lipid peroxidation.^23^^,^^24^ Recently, the roles of statins in ferroptosis have received considerable attention, particularly in cancer therapy.^24^, ^25^, ^26^, ^27^ However, some contradictory results have been reported. On the one hand, statins enhance ferroptosis, particularly in cancer cells^21^, ^22^, ^23^, ^24^, ^25^, ^26^, ^27^, ^28^ and on the other, suppressive effects of statins on ferroptosis have also been reported. ^29^^,^^30^ Glutathione peroxidase 4 (GPX4) is an enzyme that converts potentially toxic lipid hydroperoxides (L-OOH) to non-toxic lipid alcohols (L-OH) using glutathione as a cofactor.^24^ GPX4 plays a central role in the prevention of ferroptosis as the mevalonate pathway is necessary for its synthesis. Cancer cells are highly dependent on the mevalonate pathway to produce cell membrane lipids, but this makes them particularly vulnerable to ferroptosis. The inhibition of the mevalonate pathway by statins may enhance ferroptosis in therapy-resistant cancer cells, potentially improving treatment outcomes. Several clinical trials have explored this approach, but their results have been mixed.^13^ As we consider that hemin-induced cell death to be ferroptosis,^18^ the present study also supports the existence of suppressive effects with statins. Contradictory results may reflect differences in cell types, stimulators, and/or the use of statins at different doses and durations. Normal cells possess multiple pathways that prevent ferroptosis.^23^^,^^24^ Reduced GPX4 levels may be compensated for by other pathways, depending on the type of cell and the stress it experiences, which may explain discrepancies observed in terms of the effects of statins.

The lipophilicity of statins represents another important factor in this question. In Japan, six statins are available clinically; PS, atorvastatin, rosuvastatin, Pita, Sim, and Flu. PS is highly hydrophilic, and requires carrier-mediated uptake into the liver, resulting in high hepato-selectivity.^10^^,^^11^ In contrast, lipophilic statins such as atorvastatin, Sim and Flu, are capable of passive diffusion through the cell membrane and thus show significantly less hepato-selectivity.^10^^,^^11^ The low efficacy of PS observed in the present study supports the significance of lipophilicity in terms of the pleiotropic effects of statins.

In this study, DSX showed cytoprotective effects at lower concentrations than those required for the suppression of ROS and/or LIP, suggesting that DSX may exhibit cytoprotective effects independent of suppression of ROS and/or LIP, as Messa et al. previously reported.^31^

The iron chelating ability of ELT has been reported previously.^14^^,^^15^ This study further confirmed this property, as it found that ELT was able to inhibit hemin-induced increases in LIP in a dose dependent manner, and suppress cell death at concentrations as low as 1 μmol/L. Since the serum level of ELT is 10 μmol/L in the treatment of thrombocytopenia, ELT may work as an iron chelator without influencing platelet count for patients undergoing this treatment. Although ETL is currently approved only for the treatment of aplastic anemia and chronic immune thrombocytopenia, it has the potential of being used as a powerful iron chelator in the near future.

## Conclusions

This study demonstrated the suppressive effects of statins on hemin-induced cell death. Flu significantly suppressed hemin-induced ROS generation and increases in LIP, and also enhanced the suppressive effects of DSX. These results suggest that TRIO therapy may be improved by using statins in combination with low-dose DSX beginning in earlier disease stages. However, all the results of this study were obtained using the THP-1 cell line and thus further research is necessary to see whether statins will act in the same way *in vivo* and in primary human monocytes/macrophages.

## Funding

This work was supported by JSPSKAKENHI (Grant Number JP19K07181).

## Author contributions

Shion Imoto: Conceptualization, Investigation, Validation, Writing original draft. Katsuyasu Saigo: Methodology, Writing review & editing, Supervision. Mari Kono: Visualization, Investigation, Validation. Ayako Ohbuchi: Investigation, Validation. Tohru Sawamura: Investigation, Validation. Yuji Mizokoshi: Investigation, Validation. Takashi Suzuki: Investigation, Supervision.

## Conflicts of interest

There are no conflicts of interest to report.
